# Adjuvant‐induced arthritis induces epithelial proliferation and differential expression of SVS2 and SVS3 in the seminal vesicles

**DOI:** 10.1111/andr.70085

**Published:** 2025-07-07

**Authors:** Thamires Miyako Ito Sigole, Camila Reis Santos, Isabela Fiorentino Souza Nascimento, Erick J. R. Silva, Agnaldo Bruno Chies, Maria Angélica Spadella

**Affiliations:** ^1^ Graduate in Medicine Marília Medical School‐FAMEMA Marília São Paulo Brazil; ^2^ Master in Health and Aging Marília Medical School‐FAMEMA Marília São Paulo Brazil; ^3^ Department of Biophysics and Pharmacology Institute of Biosciences of Botucatu São Paulo State University Botucatu São Paulo Brazil; ^4^ Laboratory of Pharmacology Marília Medical School‐FAMEMA Marília São Paulo Brazil; ^5^ Laboratory of Human Embryology Marília Medical School‐FAMEMA Marília São Paulo Brazil

**Keywords:** adjuvant‐induced arthritis, seminal vesicle, PCNA, SVS2 and SVS3, testosterone, fertility

## Abstract

**Background:**

Rheumatoid arthritis (RA) is an inflammatory disease triggered by chronic and systemic activation of the immune system, with consequences for male fertility. However, the influence of RA on sexual accessory glands remains poorly investigated.

**Objectives:**

This study evaluated the late impact of adjuvant‐induced arthritis (AIA) on the morphophysiology of the seminal vesicles and verified whether these effects can be influenced by androgen deprivation.

**Materials and methods:**

Adult male Wistar rats were allocated into four experimental groups and subjected to induction of AIA (via injection of *Mycobacterium tuberculosis* in the right hind paw), orchiectomy (ORX), both procedures (ORX/AIA), or neither (SHAM). Forty days after AIA induction, the seminal vesicles were processed for histopathological and morphometric‐stereological analysis. Collagen deposition was measured, and immunostaining was conducted to determine PCNA, SVS2, and SVS3 expression. Serum testosterone was determined at 15 and 40 days after AIA induction.

**Results:**

Body mass and wet weight of the seminal vesicles decreased in the AIA‐induced and orchiectomized groups, and testosterone levels decreased in the AIA group. Collagen deposition in the seminal vesicle stroma increased in the orchiectomized rats, but not in the AIA rats. Induction of AIA promoted cellular proliferation in the distal region of the seminal vesicles in both intact and orchiectomized rats, and levels of two significant secretory proteins in the vesicle also changed with AIA: SVS2 increased, while SVS3 decreased. Conversely, orchiectomy decreased the expression of both SVS2 and SVS3.

**Discussion:**

The late impact of AIA on seminal vesicle parameters appears to be compensated by an increase in epithelial cell proliferation and changes in secretory activity, in an androgen‐dependent manner.

**Conclusion:**

The data suggest that transient arthritic hypoandrogenism and the direct action of joint inflammatory mediators may influence the seminal vesicle activity. Further research is required to better address the repercussions of arthritis on male fertility.

## 1 INTRODUCTION

Rheumatoid arthritis (RA) is an autoimmune disease characterized by chronic destructive inflammation, which can be influenced by genetic, epigenetic, and environmental factors in systemic and polyarticular pathways.^1^, ^2^, ^3^ It is classically typified by synovitis resulting from autoantibody formation, with joint destruction, deformity, and decreased functional capacity, impacting work and quality of life.^4^, ^5^, ^6^

The inflammatory process begins with infiltration of macrophages as well as T CD4^+^ lymphocytes, which prevail in the process and mediate production of pro‐inflammatory cytokines such as interleukin‐1 (IL1), tumor necrosis factor (TNF), interleukin‐6 (IL6), nitric oxide, and prostaglandins, components of the synovium which aggravate inflammation.^7^, ^8^ Inflammation is followed by the formation of pannus, proliferated and invasive synovial tissue that leads to progressive destruction and severe joint disability.^9^, ^10^

Studies suggest that RA can affect the male gonadal function as part of its systemic outcomes, leading to subfertility.^11^ Increased incidence of testicular dysfunction and decreased production and bioavailability of testosterone have been reported in men with RA.^12^, ^13^ Furthermore, serum testosterone and dehydroepiandrosterone sulfate (DHEAS) levels are inversely related to the RA activity, while DHEAS is also inversely correlated with disease duration and clinical severity.^10^, ^14^ On the other hand, clinical and epidemiological evidence indicates that androgens serve as protectors in men against the development of immunoinflammatory diseases, since autoimmune diseases (including RA) are more prevalent in men with untreated hypogonadism. This suggests that androgens play a role as anti‐inflammatory hormones capable of inhibiting immune, cellular, and humoral responses.^11^, ^14^, ^15^, ^16^ The androgen deficiency observed in patients with RA is likely a consequence of the disease, rather than its cause.^14^

The pathophysiological mechanisms involved in hypogonadism and consequent lower production and bioavailability of androgens are complex and not yet fully clear. Studies in arthritic rats suggest that the activation of testicular macrophages results in the production of pro‐inflammatory cytokines such as IL1 and IL6, impairing the production of testosterone.^17^, ^18^ The effect of these inflammatory mediators seems to be biphasic, decreasing production of testosterone in the Leydig cells while simultaneously promoting an increase in gonadotropins.^18^ The specific mechanisms of the anti‐inflammatory effect of testosterone have been shown to involve decreased secretion of IL1B, IL6, TNF, and other pro‐inflammatory mediators, such as monocytes and macrophages, as well as increased production of IL10 by T cells and inhibition of mediators that activate IL6 and T cell proliferation. In arthritic and orchiectomized animals, for example, androgen deficiency resulted in increased inflammation and disease activity.^19^

The adjuvant‐induced arthritis (AIA) model in rats is widely used to elucidate the pathophysiological mechanisms underlying RA.^20^, ^21^ Our research group has demonstrated histopathological changes in the testes of AIA rats, including an increase in abnormal tubule percentage and reductions in seminiferous epithelium thickness, daily sperm production, and cell proliferation. We also detected an AIA‐induced decrease in luminal density of the secretory ducts in the prostate.^22^

Preclinical investigations have not yet thoroughly explored the impact of AIA in the seminal vesicles. This knowledge gap is critical, considering the important role the seminal vesicles play in forming sperm‐nurturing semen. After ejaculation, the semen forms a viscous mass known as a semen coagulum that keeps the spermatozoa immotile, preserving their energy and promoting survival in the uterus. After liquefaction of the semen, the spermatozoa acquire progressive motility and gain fertilizing capacity after remaining in the female reproductive tract for an appropriate period (sperm capacitation). Proteins secreted by the seminal vesicles, such as seminal vesicle‐secreted protein 2 (SVS2) and SVS3, are the main components of the semen coagulum in rodents^23^; SVS2 binds the ejaculated spermatozoa, preventing premature capacitation.^23^, ^24^

In rodents, *Svs2* is an androgen‐dependent gene and a member of the REST (rapidly evolving seminal‐vesicle‐transcribed) family that comprises other *Svs* genes abundantly expressed in the seminal vesicles, including *Svs3a*, *Svs3b*, *Svs4*, and *Svs5*. The *Svs2* gene is homologous to human semenogelin‐1 (SEMG1), which is the most abundant protein in human seminal plasma and an endogenous inhibitory factor of sperm motility.^23^, ^25^ Interestingly, male *Svs2‐*null mice exhibit normal sperm production but are subfertile due to sperm cell death in the uterus, where SVS2 serves to protect the spermatozoa against uterine attack.^26^, ^27^ The effects of SVS2 on sperm capacitation are two‐fold: this protein inhibits initiation of capacitation (as a capacitation inhibitor) and cancels the fertility of the capacitated spermatozoa (as a decapacitation factor).^24^ Studies have shown that SVS3 binds both SVS2 and spermatozoa, facilitating the inhibitory effects of SVS2 on sperm capacitation.^27^, ^28^

To further investigate this phenomenon, we evaluated the late effects of AIA on the morphophysiology of seminal vesicles in rats to determine whether arthritis can influence the glandular secretory activity in androgen deprivation.

## 2 MATERIALS AND METHODS

### 2.1 Animals

Fifty‐eight adult male *Wistar* rats (350–400 g, 12 weeks old) were obtained from the animal facility at the Marilia Medical School in Marilia, SP, Brazil. The animals were maintained in polypropylene cages (50 cm × 40 cm × 20 cm, 2 rats per cage) under controlled temperature (23 ± 1°C) and lighting (12 h/12 h light–dark cycle) conditions, with ad libitum access to pelleted chow (Nuvilab CR‐1, Nuvital Nutrients, Curitiba, Brazil) and filtered water. All procedures were conducted according to the National Guidelines for Care and Use of Laboratory Animals (CONCEA) and approved by our Institutional Board on Animal Experimentation (CEUA‐FAMEMA process number 1026/14).

### 2.2 Study design and experimental groups

The rats were first subjected to orchiectomy (ORX) or sham ORX. Twenty days after surgery and rehabilitation, they received an injection to provoke AIA or a sham. One cohort of animals was studied 40 days post‐AIA induction to investigate whether potential changes in the seminal vesicles associated with arthritis‐induced hypoandrogenism (observed 15 days after immunization) are modified at a later stage of the disease, when the inflammatory process is in remission.^29^ This cohort was divided into four experimental groups: SHAM (*n* = 10), false‐orchiectomized and false‐immunized; AIA (*n* = 12), false‐orchiectomized and immunized; ORX (*n* = 10), orchiectomized and false‐immunized; and ORX/AIA (*n* = 10), orchiectomized and immunized. Solely to confirm the arthritis‐induced hypoandrogenism previously evidenced at 15 days post‐AIA in this model,^22^ a second cohort of sixteen additional rats (eight SHAM and eight AIA) was subjected to immunization or sham induction, and circulating testosterone serum levels were evaluated.

### 2.3 Orchidectomy protocol

Rats were subjected to ORX at 12 weeks of age, as described by Santos et al.^22^ Briefly, under anesthesia with 2,2,2‐tribromoethanol (25 mg/100 g; i.p.; Sigma‐Aldrich, St. Louis, MO, USA), both testes were removed through a longitudinal scrotal incision. At this age, the rats have reached sexual maturity, and testosterone production is physiologically stable, highlighting the effects of hormonal deprivation.^30^ False‐orchiectomized rats were subjected to the same surgical procedure, except the testes were preserved. The effectiveness of orchidectomy was confirmed by undetectable levels of testosterone in serum harvested 40 days post‐AIA induction.

### 2.4 Adjuvant‐induced arthritis protocol

For AIA induction, the animals were anesthetized with tribromoethanol (25 mg/100 g; i.p.; Sigma‐Aldrich, St. Louis, MO, USA) and positioned in ventral decubitus on the surgical field. Arthritis was induced by intradermal injection of 100 µL of emulsion containing a mixture of mineral oil and distilled water and 3.8 mg/mL of heat‐inactivated *Mycobacterium tuberculosis* strain H37Ra (Becton, Dickinson and Company, Sparks, MD, USA) in the right hind paw. False‐immunized rats underwent the same procedure but received only mineral oil. In the post‐induction period, the effectiveness of AIA induction was monitored daily by ectoscopy of the paws, and confirmed by the presence of inflammatory signs in the hind paw contralateral to the induction (usually observed around 15–20 days after immunization).

### 2.5 Sample collection

Forty days after AIA induction, animals were weighed, anesthetized with 2,2,2‐tribromoethanol (25 mg/100 g; i.p.; Sigma‐Aldrich, St. Louis, MO, USA), and euthanized by exsanguination via puncture of the inferior vena cava. The seminal vesicles with fluid were dissected, and the wet mass was determined. Left hind paws were dissected just before the tibiotarsal joint, and weighed to confirm articular inflammation. Values for wet weight of the seminal vesicles (g) were normalized according to the respective body mass (kg) of each rat studied.

### 2.6 Determining serum testosterone

Blood samples were collected via inferior vena cava puncture immediately after euthanasia at 15 or 40 days after AIA induction (according to each group), using BD Vacutainer blood collection tubes (Serum [Ref 367820], Becton, Dickinson and Company, Sparks, MD, USA) to measure serum testosterone levels. The harvested blood samples were centrifuged for 20 min at 4°C (1917 ×*g*) to obtain serum, which was stored in a freezer at −80°C until testosterone levels were determined using the chemiluminescence method at a private veterinary laboratory. The serum testosterone level was expressed in ng/dL.

### 2.7 Histopathological analysis

The seminal vesicles were fixed in 4% paraformaldehyde (MERK, Darmstadt, Germany) in phosphate‐buffered saline (PBS), pH 7.2, for 24 h. Afterward, they were washed in running water for 24 h and maintained in 70% ethyl alcohol until further processing. The samples were then dehydrated in a series of increasing alcohol concentrations, diaphanized in butyl alcohol, infiltrated, and embedded in Paraplast Plus tissue embedding medium (McCormick Scientific, St. Louis, MO, USA). The 5‐µm‐thick sections were obtained using a steel blade and subsequently subjected to hematoxylin–eosin (HE) staining for histopathological analysis of epithelium, stroma, and lumen according to Santos et al.^22^ For each animal, slides containing two non‐serial sections were analyzed. Micrographs were obtained using an Olympus DP‐25 digital camera attached to the Olympus BX41 microscope, and CellSens by Dimension image capture software (Olympus, Tokyo, Japan) was utilized.

### 2.8 Total collagen evaluation

For the analyses, 5‐µm‐thick sections in Paraplast Plus tissue embedding medium (McCormick Scientific, St. Louis, MO, USA) were examined utilizing the picrosirius red histochemical method.^31^, ^32^ A mean of ten histological fields of the distal and intermediary regions of the gland per rat were randomly captured under polarized light using an Olympus DP‐25 digital camera attached to the Olympus BX41 microscope. To quantify total collagen, the number of points coincident with the areas of interest (*n*) was determined with a manual threshold adjustment tool in the Olympus CellSens by Dimension software (Olympus, Tokyo, Japan).

### 2.9 Morphometric‐stereological analysis

At least 10 histological fields of the intermediary region of each gland were randomly captured from two histological sections with HE staining. From these captured fields, the height (µm) of the glandular epithelium was measured using a linear line tool in CellSens Standard image analysis software (Olympus, Tokyo, Japan); this region was chosen as representative of the gland activity.^33^ In these same fields, the density (*D*) of the lumen (Lu), epithelium (Ep), and stroma (St) in each seminal vesicle was obtained using a grid according to Weibel^34^ with 156 intersections distributed in geometrically equal areas. The intermediary region of each gland was then evaluated by projecting the grid onto the histological field, counting the number of coincident points (*n*) in these regions. The densities occupied by each component were calculated by the formulas:

$$D\;\left(\mathrm{Lu}\right)=\frac{\mathrm{total}\;\mathrm{number}\;\mathrm{of}\;\mathrm{Lu}\;\mathrm{counted}}{156}\;\;.\;100$$

$$D\;\left(\mathrm{Ep}\right)=\frac{\mathrm{total}\;\mathrm{number}\;\mathrm{of}\;\mathrm{Ep}\;\mathrm{counted}}{156}\;.\;100$$

$$D\left(\mathrm{St}\right)=\frac{\mathrm{total}\;\mathrm{number}\;\mathrm{of}\;\mathrm{St}\;\mathrm{counted}}{156}.100$$

### 2.10 Immunohistochemistry assays for PCNA, SVS2, and SVS3

First, 5‐µm‐thick sections of the seminal vesicles were deparaffinized in xylene and rehydrated in graded ethanol solutions, then dried in an oven at 60°C for 60 min. Next, antigen retrieval was performed by placing the sections in a microwave oven at 700–800 W for 15 min (3 cycles of 5 min) in 0.01 M pH 6.0 sodium citrate buffer; they were then blocked with 3% hydrogen peroxide in methanol for 30 min. The slides were incubated in a 3% Molico milk solution (Nestle, São Paulo, Brazil) for 1 h to block nonspecific binding. They were then incubated overnight with the primary antibodies in a humid chamber at 4°C (rat anti‐PCNA antibody [PC10] [Ab29] [Abcam Inc., Cambridge, UK] at 1:300; rat anti‐SVS2 [epitope: R^352^KNFNPGNYFTKGGADL^368^ of mouse SVS2] and anti‐SVS3 antibodies [epitope: D^75^ADADMGGALSSQE^88^ of mouse SVS3B] [GenScript Inc., Piscataway, New Jersey, USA] at 1:300 and 1:300, respectively; the anti‐SVS3 antibody recognizes both SVS3 A and SVS3 B isoforms). The slides were then washed with PBS and incubated with Polymer N‐Histofine RAT Multi reagent (Nichirei Biosciences Inc., Tokyo, Japan) for 30 min at room temperature. They were washed again in PBS and subjected to diaminobenzidine (DAB) for 3 min, and subsequently counterstained with hematoxylin. Positive and negative controls were obtained. To quantify the positive cells, 10 histological fields of the seminal vesicles per animal were captured (distal and proximal regions for PCNA and distal and intermediary regions for SVS2/SVS3), using an Olympus DP‐25 digital camera attached to an Olympus BX41 microscope (Olympus, Tokyo, Japan). The number of labeled points was counted using a manual threshold adjustment tool in Olympus CellSens by Dimension software (Olympus, Tokyo, Japan), as proposed by Santos et al.^22^

### 2.11 Statistical analysis

Data normality was verified according to the Kolmogorov–Smirnov test, and the homogeneity of variances was tested using the Levene test. One‐way ANOVA was used to compare means among the groups, followed by the Bonferroni post hoc test. For data that violated the homogeneity of the variances, the robust Welch test was used to compare the means among the groups, followed by the Games–Howell post hoc test. For comparison between two independent groups, the Kruskal–Wallis non‐parametric test was used for data without normal distribution, followed by the Mann–Whitney test for pair‐to‐pair comparisons. Parametric data were expressed as mean ± standard deviation (SD), while non‐parametric data were expressed as median (Min to Max). Values of *P* < 0.05 were considered statistically significant. The analyses were performed using SPSS software (IBM Corporation, Armonk, NY, USA), version 19.0.

## 3 Results

### 3.1 Arthritis and orchiectomy promote a decrease in body mass and wet weight of the seminal vesicles

At 40 days post‐induction, AIA and ORX promoted a significant decrease in body weight in the animals that underwent these procedures compared to those in the sham group. The wet mass of the seminal vesicles significantly decreased in the AIA, ORX, and ORX/AIA groups, with notable impacts from arthritis induction and ORX (Table 1). A sharp reduction was seen in wet mass of the glands in the ORX and ORX/AIA groups, demonstrating the effectiveness of ORX, while wet mass of the left hind paw (contralateral to the paw that underwent induction) increased significantly in the AIA and ORX/AIA groups as a result of AIA, confirming the effectiveness of the model and that the disease reached a systemic level (Table 1).

**TABLE 1:** Biometric parameters in each experimental group.

Parameters	SHAM (*n* = 10)	AIA (*n* = 12)	ORX (*n* = 10)	ORX/AIA (*n* = 10)	*p*‐value
Body weight (g)	441.5 ± 35^€^, ^‡^, ^†^	349.0 ± 36	384.0 ± 27	338.0 ± 60	0.0001^*^
Left hind paw (g)	5.04 ± 0.56^€^, ^†^	9.27 ± 3.50^‡^	5.44 ± 0.33^†^	10.84 ± 5.25	0.0001^**^
Seminal vesicle (g)	3.53 ± 0.85^€^, ^‡^, ^†^	2.52 ± 0.78^‡^, ^†^	0.32 ± 0.15	0.38 ± 0.11	0.0001^**^
Seminal vesicle (g/kg)	8.10 ± 1.95^‡^, ^†^	7.17 ± 1.80^‡^, ^†^	0.84 ± 0.40	1.21 ± 0.63	0.0001^**^

*Note*: Data are expressed as mean ± SD.

Abbreviations: ORX, orchiectomy; AIA, adjuvant‐induced arthritis.

^*^ *P *< 0.05 Anova‐one‐way followed by the Bonferroni test;

** *P *< 0.05 Welch test followed by Games–Howell test.

^€^ significant difference to AIA group;

^‡^ *P *< 0.05 significant difference to ORX group;

^†^ *P* < 0.05 significant difference to ORX/AIA group. In parentheses, the number of rats per group. g = grams. g/kg, wet weight (g) per kg body weight.

### 3.2 The adjuvant‐induced arthritis‐induced decrease in testosterone levels self‐limits 40 days after immunization

At 15 days after AIA induction, testosterone levels significantly decreased in the arthritic rats. Although the AIA animals also showed a drop in serum testosterone levels 40 days post‐induction, no significant difference was found compared to the SHAM group. The testosterone levels in the ORX and ORX/AIA rats were reduced to undetectable values, reinforcing the effectiveness of ORX (Table 2).

**TABLE 2:** Serum testosterone levels among the experimental groups at 15 and 40 days after AIA induction.

Testosterone (ng/mL)	SHAM	AIA	ORX	ORX/AIA	*p*‐value
15 days	260.4 ± 80.67 (*n* = 8)	11.50 ± 2.27* (*n* = 8)	–	–	0.0002
40 days	205.40 ± 164.90 (*n* = 10)	148.34 ± 84.72 (*n* = 12)	ND (*n* = 10)	ND (*n* = 10)	0.147

*Note*: Data are expressed as mean ± SD.

Abbreviations: ORX, orchiectomy; AIA, adjuvant‐induced arthritis.

*P *< 0.05, the Kruskal–Wallis non‐parametric test, followed by the Mann–Whitney test for pair‐to‐pair comparisons. * Significant difference in relation to the SHAM group. In parentheses, the number of independent determinations. “–” = non‐determined; ND, non‐detectable values, therefore not considered in the statistical analysis.

### 3.3 Androgen deprivation (alone or associated with arthritis) alters the morphology of the seminal vesicles

**FIGURE 1:**
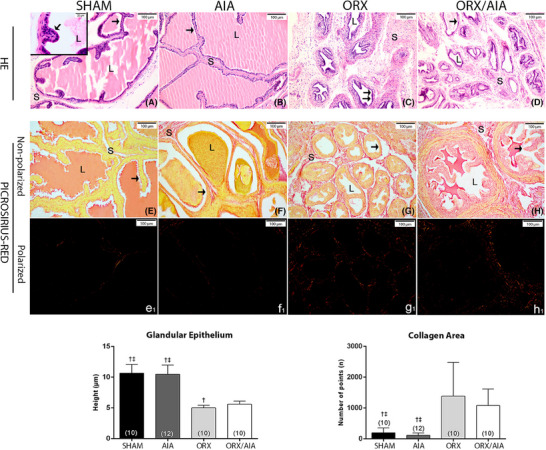
Representative photomicrographs of the seminal vesicles from SHAM (A, E, e_1_), AIA (B, F, f_1_), ORX (C, G, g_1_), and ORX/AIA groups (D, H, h_1_). HE sections show in the SHAM and AIA groups that gland ducts are lined by columnar secretory epithelial cells (arrow). Note the lumen (L) occupied by secretion and the thin stroma (S) around the ducts. In the ORX and ORX/AIA rats, the ducts have a cuboidal epithelium (arrow), with a lumen reduction and an increase in the stromal area. Picrosirius‐red sections under non‐ and polarized light show the total collagen labeled in red in the stroma. Morphometry data of the glandular epithelium and collagen area are presented. Legend: Data are expressed as mean ± SD. Data were analyzed using the Welch test, followed by the Games–Howell test. † *P* < 0.05 significant difference to ORX/AIA group; ‡ *P* < 0.05 significant difference to ORX group. In parentheses, the number of independent determinations. HE, Hematoxylin–Eosin. Scale bar = 50 µm (magnification 400×); inset = 20 µm (magnification 1000×). ORX, orchiectomy; AIA, adjuvant‐induced arthritis.

The rats in the SHAM and AIA groups exhibited seminal vesicles with standard morphology 40 days after induction, containing numerous secretory ducts lined with a typical columnar epithelium and a lumen filled with acidophilic secretion. A narrowband of stroma consisting of smooth muscle cells and loose connective tissue was observed interspersing the ducts (Figure 1A,B). In contrast, the glands in the ORX and ORX/AIA groups suffered intense structural modification due to the drastic drop in testosterone levels caused by ORX. The epithelium lining the ducts was cuboid in appearance, with reduced or scarce secretion in the lumen and enlargement of the stromal area (Figure 1C,D). The morphometric data corroborated the histopathological findings, showing a significant decrease in seminal vesicle epithelium height in the ORX and ORX/AIA groups; epithelial height of the seminal vesicle ducts was particularly lower in the ORX group compared to the ORQ/AIA group. Arthritis was not found to significantly affect glandular epithelium height (Figure 1).

### 3.4 Adjuvant‐induced arthritis does not change the deposition of collagen in the stroma of the seminal vesicles

Assessment of total collagen from the seminal vesicles in the experimental groups 40 days post‐induction found an increase in collagen fibers in the stroma surrounding the atrophied secretory ducts in the ORX and ORX/AIA animals compared to the SHAM and AIA groups (Figure 1e–h, e_1_–h_1_). The collagen count data confirmed this significant increase in collagen in the glandular stroma of the ORX and ORX/AIA groups. Alterations in collagen deposits were not seen in the stroma of the AIA rats (Figure 1).

### 3.5 Orchiectomy induces alterations in luminal and stromal densities of the seminal vesicles that are not observed in adjuvant‐induced arthritis

**FIGURE 2:**
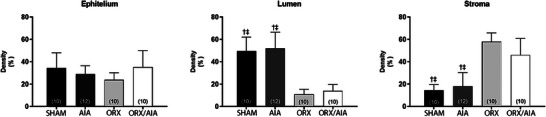
Densities of epithelium, lumen, and stroma of the seminal vesicles in each experimental group.data are expressed as mean ± SD. Data on luminal density were analyzed by one‐way ANOVA, followed by the Bonferroni test; data on stroma density were analyzed using the Welch test, followed by the Games–Howell test. † *P* < 0.05 significant difference to ORX/AIA group; ‡ *P* < 0.05 significant difference to ORX group. In parentheses, the number of independent determinations. ORX, orchiectomy; AIA, adjuvant‐induced arthritis.

Neither arthritis nor ORX affected epithelial density in the seminal vesicles of the rats. On the other hand, significant reduction in the lumen and increased stromal volume were found in the seminal vesicles of the ORX and ORX/AIA groups compared to the animals in the SHAM and AIA groups (Figure 2).

### 3.6 Adjuvant‐induced arthritis influences cellular proliferation in the distal region of the seminal vesicles

**FIGURE 3:**
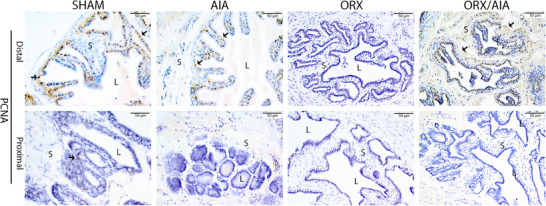
Representative photomicrographs of PCNA immunohistochemistry of the seminal vesicles among the experimental groups, exhibiting the distal and proximal regions. Positive immunostaining is observed in the nuclei of the epithelial cells surrounding the ducts. Note in the orchiectomized group, an absence of marking in the cells. Legend: immunoperoxidase staining is brown (arrowhead). Counterstained with hematoxylin. L = lumen; S = stroma. Scale bar = 50 µm (magnification 400×).

At 40 days post‐induction, a positive reaction for PCNA was found in the seminal vesicles of the SHAM and AIA rats in nuclei of luminal and basal cells in the distal region, denoting proliferation of the epithelial cells. In the ORX group, non‐positive cells were observed in the distal region of the gland, while a higher number of marked cells were found in this region in the seminal vesicles of the ORX/AIA animals. Rare or non‐PCNA‐positive cells were observed in the proximal region of the gland, with no significant difference among the experimental groups. Comparison of immunostaining in both regions within the same group demonstrated a significant reduction in the number of marked cells in the proximal region (Figure 3, Table 3).

**TABLE 3:** Quantification of PCNA‐, SVS2‐, and SVS3‐positive cells in the experimental groups.

Positive‐labeled	Mean	SD	Median	Min	Max	*p*‐value
PCNA						
[dis]						
SHAM	436.2	181	513	134	575	0.007^*^
AIA	185.6	108.4	227	0	263	
ORX	0^‡^, ^†^	0	0^‡^, ^†^	0	0	
ORX/AIA	114^‡^	119.1	100^‡^	0	260	
[pro]						
SHAM	0.14	0.47	0	0	2	0.41
AIA	0.29	0.86	0	0	3	
ORX	0.23	0.6	0	0	2	
ORX/AIA	0	0	0	0	0	
SVS2						
[dis]						
SHAM	2545	1641	2136	0	5817	<0.001^*^
AIA	6793^‡^	3862	6381^‡^	0	16393	
ORX	1133^‡^, ^†^	2203	0^‡^, ^†^	0	7776	
ORX/AIA	931^‡^, ^†^	2181	0^‡^, ^†^	0	10100	
[int]						
SHAM	917	959	766	0	3205	<0.001^*^
AIA	2404^‡^	2357	1556^‡^	0	6824	
ORX	0^‡^, ^†^	0	0^‡^, ^†^	0	0	
ORX/AIA	1679	3065	0^†^	0	7746	
SVS3						
[dis]						
SHAM	12638	10368	8206	0	29406	<0.001^*^
AIA	4715^‡^	6338	2012^‡^	0	31155	
ORX	1262^‡^, ^†^	2723	0^‡^, ^†^	0	11623	
ORX/AIA	644^‡^, ^†^	777	224^‡^, ^†^	0	2497	
[int]						
SHAM	2299	2108	1804	0	7100	<0.001^*^
AIA	1173^‡^	1467	586^‡^	0	4904	
ORX	1233^‡^, ^†^	2123	0^‡^, ^†^	0	8865	
ORX/AIA	624^‡^, ^†^	873	0^‡^, ^†^	0	2927	

Abbreviations: ORX, orchiectomy; AIA, adjuvant‐induced arthritis.

* *P *< 0.05 significant difference between groups by the Kruskal–Wallis test.

^‡^ *P* < 0.05 significant difference to the SHAM group;

^†^ *P* < 0.05 significant difference to the AIA group. In parentheses, the number of animals per group. pro = proximal area; dis = distal area; int = intermediary area.

### 3.7 Adjuvant‐induced arthritis increases the abundance of SVS2 immunostaining, but not SVS3

**FIGURE 4:**
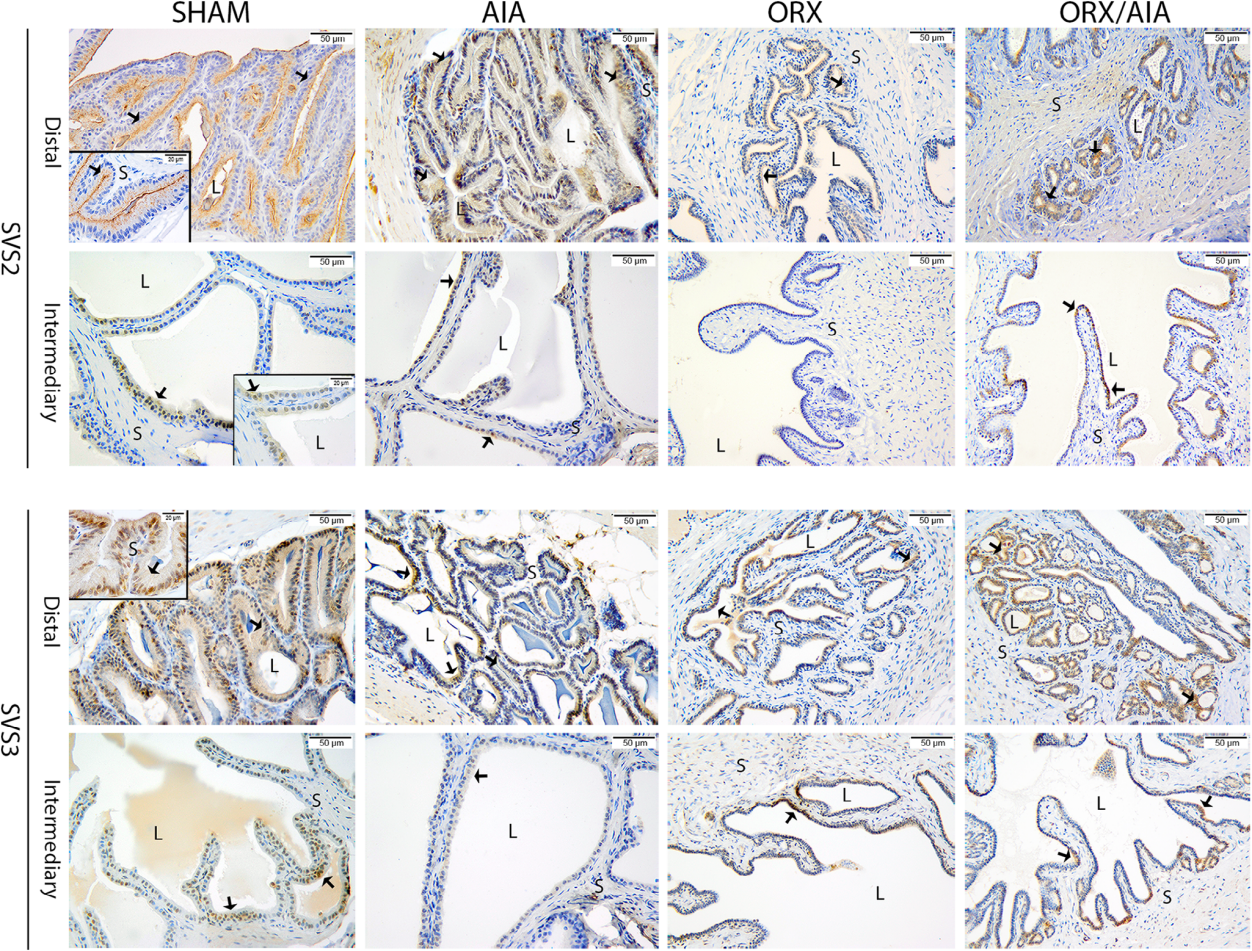
Representative photomicrographs of seminal‐vesicle secreted proteins SVS2 and SVS3 immunohistochemistry among the experimental groups, with evidence for the distal and intermediary regions. Positive immunostaining is observed in the apical pole of the cells (arrowhead) surrounding the glandular ducts. Legend: Immunoperoxidase staining is brown (arrowhead). Counterstained with hematoxylin. L = lumen; S = stroma. Scale bar = 50 µm (magnification 400×); inset = 20 µm (magnification 1000×).

In the rats assessed 40 days after AIA induction and ORX, SVS2 and SVS3 immunostaining in the seminal vesicles was observed in the apical pole of the cells lining the secretory ducts in the distal and intermediary regions of the gland. A significant increase in the abundance of SVS2‐positive immunostaining was seen in the AIA group, in both the distal and intermediary regions of the seminal vesicle (Figure 4). In contrast, SVS2‐positive immunostaining decreased in the distal region in the ORX and ORX/AIA groups compared to the SHAM and AIA groups (Figure 4). In the intermediary region, non‐labeling was observed in the seminal vesicles of the orchiectomized rats, although a reduced level of SVS2‐positive immunostaining was observed in the AIA group (Figure 4). For SVS3, significant reduction of immunostaining was observed in the distal and intermediary regions of the glands in the AIA, ORX, and ORX/AIA groups (Figure 4, Table 3).

## 4 DISCUSSION

This study investigated the late effects of AIA on the seminal vesicles of rats to determine whether these effects result from hypoandrogenism secondary to arthritis or may also reflect the direct action of inflammatory mediators on the glandular tissue. For this purpose, glands from orchiectomized rats were also examined. The AIA model used in this study, which experimentally corresponds to RA in humans, is commonly employed in research on how modulation of the immune system may affect the reproductive system.^22^, ^35^

Our findings revealed that AIA led to a decrease in the rats’ body weight, which suggests sarcopenia and may be related to metabolic changes and increased musculoskeletal proteolysis. This has clinical relevance, since humans with RA may experience rheumatoid cachexia.^36^, ^37^ Another manifestation observed in the rat AIA model was the increased mass of the paw contralateral to the induced arthritis; this suggests edema characteristic of the polyarticular inflammatory reaction that is typical in the arthritic model used.

The significant reduction in body weight observed in the ORX and ORX/AIA rats following ORX is likely a consequence of the sharp drop in testosterone. This hormonal change would lead to a decrease in lean mass, consistent with testosterone's anabolic effects.^38^ Although the difference in body mass reduction between these two groups was not significant, a greater decrease in mass was observed in the ORX/AIA animals, which may indicate that more lean mass is lost due to AIA and perhaps suggesting that the sarcopenia resulting from arthritis may be more severe in cases of hypoandrogenism. We also observed that AIA provoked a reduction in the wet weight of the seminal vesicles. As mentioned earlier, in vitro studies suggest that testicular macrophages from AIA animals secrete pro‐inflammatory mediators that act on Leydig cells, reducing testosterone production.^18^, ^39^ The observed effect of AIA on seminal vesicle mass suggests that arthritis, either directly or indirectly through hypoandrogenism caused by the inflammatory process, impacts these androgen‐dependent organs.

The effects of ORX‐induced androgen deprivation on the seminal vesicles were readily visible in the drastic reduction of wet mass. This is corroborated by descriptions in the literature of the rapid and extensive involution of these glands with decreased secretory activity, apoptotic death of epithelial cells, and stromal atrophy in rats subjected to ORX.^33^, ^40^ The reductions in body mass as well as seminal vesicle mass we observed in the ORX and ORX/AIA rats demonstrate the effectiveness of the protocol.

Serum testosterone levels in the AIA animals were lower 40 days after induction, but the difference compared to the SHAM group was not found to be significant. However, 15 days after immunization, we verified a significant decrease in testosterone levels, as also confirmed in our previous study,^22^ which confirms hypoandrogenism during this period. Considering that the onset of phlogistic signs in the AIA experimental model is rapid, of self‐limited duration, and may occur early in the model,^41^, ^42^ it is possible that at 40 days after induction, the AIA rats in this present study were in a resolutive phase, with remission of the inflammatory mediators.^22^, ^29^ In this case, the impact of AIA on testicular steroidogenesis would be transitory.

However, Asirvatham and Bruot^39^ suggested that the severity of arthritis symptoms in rodents can be inversely related to testosterone levels. In humans, the degree of the RA activity can also lead to changes in the hypothalamic–pituitary axis and consequently cause periods of gonadal dysfunction when in the clinically manifested phase. Reinforcing this hypothesis, some studies in men with RA did not find any abnormalities in the levels of androgens in some individuals; in others, a decrease was found in testosterone levels that was negatively associated with disease activity.^13^, ^19^

Our results also demonstrated that long‐lasting androgen deprivation resulting from ORX (either alone or when associated with AIA) significantly affected the morphology of the seminal glands, characterized by atrophy of the secretory ducts, increased quantities of collagen deposited in the stroma as well as enlargement of this structure, and caused a decrease in the lumen of the ducts. On the other hand, these alterations were not observed in the seminal vesicles of the non‐orchiectomized arthritic rats. These findings suggest that the articular inflammatory process may have more direct repercussions on the seminal vesicles than those related to AIA‐induced hypoandrogenism. Our previous data for the ventral prostate of both intact and orchiectomized arthritic rats corroborate this hypothesis.^22^ It is important to emphasize that the hypoandrogenism induced by arthritis may be a gradual process that limits spermatogenesis, hinders daily production of spermatozoa (as previously verified in rat testes 40 days after induction), and causes secretory dysfunctions of the male sexual glands,^22^ which can affect fertility. As a result, our findings warrant further investigation to pinpoint how androgens contribute to the effects of AIA in male accessory organs.

In the immunohistochemistry data for PCNA, an antigen used as a marker of proliferative activity in cells, we observed elevated epithelial proliferation in the distal seminal vesicles of the sham and arthritic rats, as well as those in the ORX/AIA group. Inversely, ORX disrupted cellular proliferation of the gland, which was expected. These data indicate that the inflammatory mediators delivered by AIA can induce the cellular routine of proliferation in the seminal epithelium, since both the AIA group and especially the ORX/AIA group presented PCNA‐positive cells. In the prostate, experimental and human studies have shown that immunoinflammatory factors can play an important role in the growth and proliferation processes of the epithelium, probably through modulation of a similar effect may have occurred in the seminal vesicles, suggesting AIA has a hyperplastic action on the epithelium due to the inflammatory process and may even overcome the atrophic effect of androgen deprivation in orchiectomized and arthritic animals.cytokines.^43^, ^44^, ^45^

As for SVS2, in both the distal and intermediary regions of the gland, we observed more positive immunostaining in AIA rats compared to those in the SHAM, ORX, and ORX/AIA groups. For SVS3, both regions of the seminal vesicles showed a significant reduction in all the experimental groups. In the animals deprived of androgen, this reduction was even higher, with the AIA animals (particularly those subjected to ORX and consequent hypoandrogenism) showing reduced secretion of SVS3 protein in the seminal vesicle. The divergent expression patterns for SVS2 and SVS3 could be associated with their differential sensitivities to hormonal changes within the seminal vesicles under the conditions involved with the AIA and ORX/AIA models.

Notably, SVS2 is sufficient and necessary to prevent premature capacitation and promote sperm survival in the uterus,^23^, ^26^ while SVS3 is unable to promote such effects by itself, but instead boosts the effects of SVS2 on modulation of the sperm function.^27^ The presence of arthritis leads to changes in the composition of the seminal plasma by affecting the secretion of REST proteins, which can influence sperm capacitation and function. These results allow us to hypothesize that the seminal secretory activity may be modulated by the pro‐inflammatory factors of arthritis, and the increase in SVS2 expression may be a mechanism to compensate for the negative effects of AIA on the seminal vesicles. Additional experiments are required to test this hypothesis.

In conclusion, our findings indicate that arthritis decreases the wet weight of the seminal vesicles without histological changes. However, AIA induced proliferation of the seminal epithelium and the opposite expression of SVS2 and SVS3, implying that semen composition is affected by this disease. The data suggest that both transient arthritic hypoandrogenism and articular pro‐inflammatory mediators may have a significant influence on the seminal vesicle activity.

## AUTHOR CONTRIBUTIONS

Thamires Miyako Ito Sigole performed the analyses, organized the data, and contributed to drafting the paper. Camila Reis Santos performed the orchiectomy and arthritic induction procedures in the rats. Isabela Fiorentino Souza Nascimento reviewed the images and assisted with the testosterone assays. Agnaldo Bruno Chies designed the research study and contributed to drafting the paper. Erick J. R. Silva contributed to the data analysis and drafting, and revising the paper. Maria Angélica Spadella coordinated the research, designed the research study, analyzed the data, and wrote and revised the paper.

## CONFLICT OF INTEREST STATEMENT

The authors declare no conflicts of interest.

## Supporting information




Supporting information
