# Supplementary figures and images for: Adjuvant‐induced arthritis induces epithelial proliferation and differential expression of SVS2 and SVS3 in the seminal vesicles
Source: Andrology. 2025 Jul 7;14(2):516–27. doi: 10.1111/andr.70085 (PMC12842856; doi:10.1111/andr.70085)

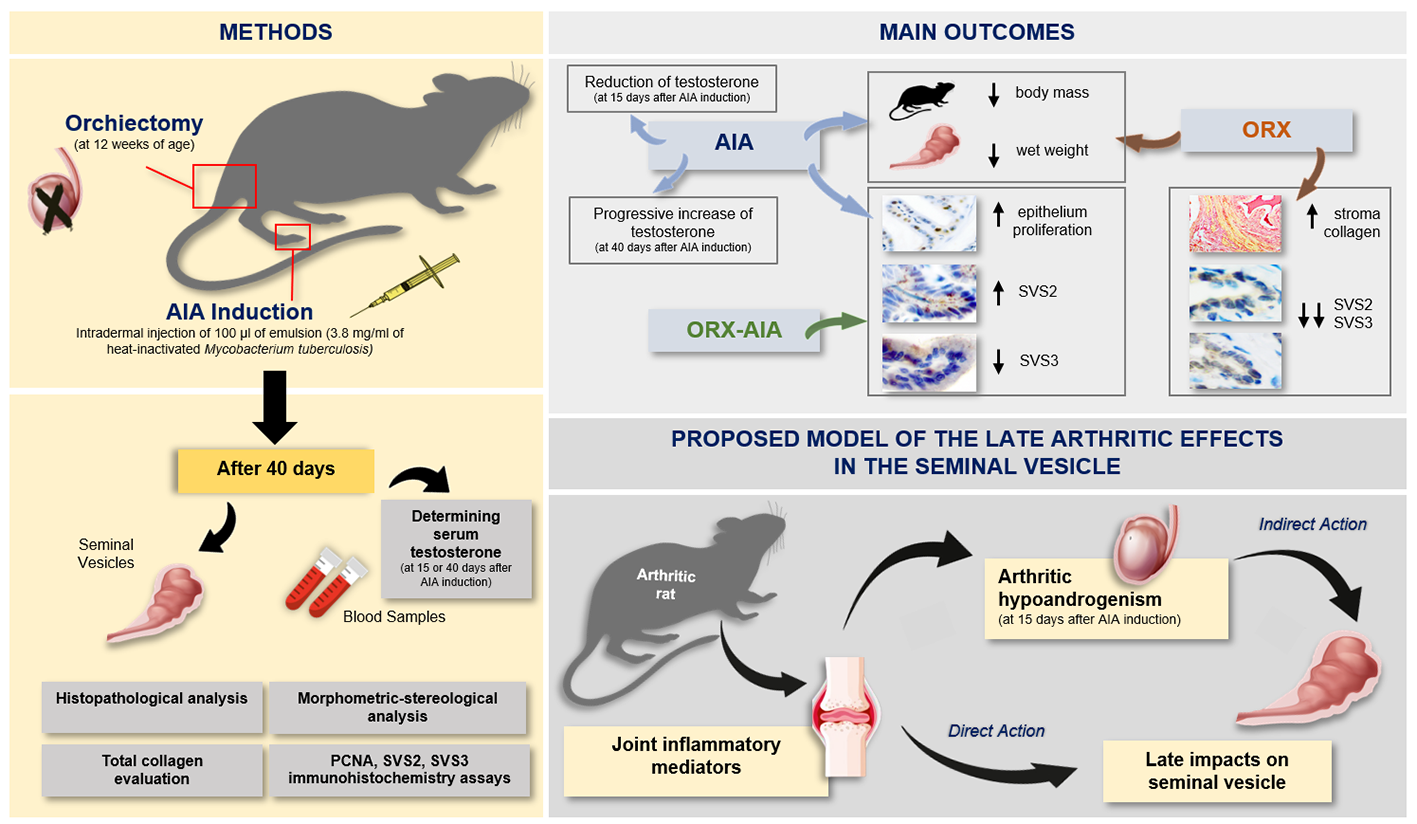

Supplement: Supplementary file 1 — Supporting information [file ANDR-14-516-s001.tif]
